# Closing the loop: establishing an autonomous test-learn cycle to optimize induction of bacterial systems using a robotic platform

**DOI:** 10.3389/fbioe.2024.1528224

**Published:** 2025-01-22

**Authors:** Jan Benedict Spannenkrebs, Aron Eiermann, Thomas Zoll, Silke Hackenschmidt, Johannes Kabisch

**Affiliations:** ^1^ Institute for Biotechnology and Food Science, NTNU, Trondheim, Norway; ^2^ Proteineer GmbH, Neu-Isenburg, Germany; ^3^ Computer-Aided Synthetic Biology, TU Darmstadt, Darmstadt, Germany

**Keywords:** automation, synthetic biology, design-build-test-learn (DBTL) cycle, autonomous system, learning algorithm

## Abstract

One goal of synthetic biology is to provide well-characterised biological parts that behave predictably in genetic assemblies. To achieve this, each part must be characterised in a time-resolved manner under relevant conditions. Robotic platforms can be used to automate this task and provide sufficiently large and reproducible data sets including provenance. Although robotics can significantly speed up the data collection process, the collation and analysis of the resulting data, needed to reprogram and refine workflows for future iterations, is often a manual process. As a result, even in times of rapidly advancing artificial intelligence, the common design-build-test-learn (DBTL) cycle is still not circular without human intervention. To move towards fully automated DBTL cycles, we developed a software framework to enable a robotic platform to autonomously adjust test parameters. This interdisciplinary work between computer science and biology thus transforms a static robotic platform into a dynamic one. The software framework includes software components such as an importer that retrieves measurement data from the platform’s devices and writes it to a database. This is followed by an optimizer that selects the next measurement points based on a balance between exploration and exploitation. The platform is shown to be able to automatically and autonomously optimize the inducer concentration for a *Bacillus subtilis* system and the combination of inducer and feed release for a *Escherichia coli* system. As a target product the readily measurable green fluorescent reporter protein (GFP) is produced over multiple, consecutive iterations of testing. An evaluation of chosen (learning) algorithms for single and dual factor optimization was performed. In this article, we share the lessons learned from the development, implementation and execution of this automated design-build-test-learn cycles on a robotic platform.

## 1 Introduction

The complexity of heterologous protein production necessitates the careful selection of inducer concentrations and growth conditions for each protein and organism, posing a multidimensional optimization challenge, with the aim of an efficient and high-yield expression (Ahmad et al., 2018). Bacterial expression systems commonly consist of a specific microbial strain and an often synthetic genetic circuit, which at the simplest level consists of a gene encoding a (heterologous) protein of interest under the control of a promoter. This promoter is often chosen to be inducible, in order to control the starting point of protein synthesis, for example, once a desired cell density is reached. A multitude of factors influence the successful production of a target protein. Major variables are the quantity of inducer, induction timepoint and media composition in order to correctly balance out process time needed, cost associated to the often expensive inducers and the amount of protein produced to maximize profit.

Over the last years, the concept of machine learning has seen rising interest and use in the fields of biology and biotechnology to address such multidimensional problems. It is used to analyse large amounts of generated data by fitting a model to the data, which will then allow predictions on the systems behavior under different conditions. By using this knowledge, future experiments can be conducted in a more targeted manner. In the field of biotechnology, machine learning can, for example, be used to find optimal growth conditions or optimal conditions for protein expression. Generated data can then be used to compliment the existing data and refine the model, starting the cycle of experiment → data measurement → model generation → prediction → experimental design → experiment again (Greener et al., 2022). One of the main constraints when using machine learning in a biological setting is the need for large enough datasets, contrasted by the fact that biological experiments are often characterized by slow speed of data generation.

To generate the amounts of reproducible data needed to efficiently use machine learning approaches, automating biological workflows is necessary. Carbonell et al. (2019) To achieve higher number of cultivations and measurements in parallel, small scale cultivation plates can be used. These can, for example, take the form of microfluidics (Husser et al., 2018), or cultivation in microtiter plates, which can be handled by robots in automated workflows. This automation leads to a significantly higher throughput and faster turnaround compared to a human experimenter (Torres-Acosta et al., 2022). Traditionally, such optimizations involve prolonged cycles of ‘Design-Build-Test-Learn’ and large amounts of manual labor to generate, curate and interpret the gathered data. Complicating matters further, biological variability and batch-to-batch differences introduce potential sources of noise, making data analysis challenging and increasing the risk of false results (Bähner et al., 2021). In 2016, Keasling et al. introduced the concept of seamlessly integrating machine learning software with liquid handling robots, offering a fully automated approach to optimization, eliminating the need for human intervention (Nielsen and Keasling, 2016). By combining lab automation and machine learning, shorter experiment (turnover) and data analysis times are achieved (Carbonell et al., 2019). This fast turnaround results in a fully automated continuous optimization, determining new points of interest in a generated model after a sequential cultivation has already started. The model generated from gathered data factors in an equilibrium between exploration and exploitation (Thompson et al., 2023), identifying areas of interest to be screened for the next experiment. This is contrasted by the long time necessary for data interpretation without machine learning (HamediRad et al., 2019). In recent years, the use of robotic platforms in a biological research context has gained broader popularity, changing from pure liquid handling platforms to integrated platforms capable of starting, cultivating and measuring bacterial cultures without the need for human interference. Such concepts found use, for example, in bioprocesses Nickel et al. (2017) and a lycopene biosynthetic pathway optimization, another microbiological problem that tries to optimize a microbiological system not by using the best inducer concentrations but by exchanging the microbiological switches HamediRad et al. (2019). Wollerton *et al.* demonstrated a platform capable of autonomous cultivation of bacteria, as well as lysis of the cultures and protein purification (Wollerton et al., 2006). Multiple papers have also pointed out the challenges associated with high throughput biological experiments using robotic platforms, such as interoperability of components and coordinating the different, often simultaneously occurring tasks by using a form of scheduler system to keep track of operations performed and labware moved (Kaspersetz et al., 2024; Casas et al., 2024). While the combined use of machine learning and automated robotic platform has shown promising results for optimization, utilizing multiple iterations of the DBTL cycle, for example, for the production of dodecanol by *E.coli*, (Opgenorth et al., 2019) commonly human intervention between iterations is still required. In this study we present our findings gathered from integrating different optimization algorithms on a robotics platform to maximize protein production for two biological systems. An active-learning approach, utilizing machine learning and a random search algorithm are used to seek out the optimal solution within a multi-dimensional matrix. The input variables being the amount of inducer (lactose/IPTG) added, as well as the amount of enzyme added, releasing glucose from a polysaccharide, and thus allowing control of growth rates. The measured output variables are fluorescence, caused by GFP production as well as cell density (OD_600 nm_). The platform then analyses the gathered data and directly uses it for the next round of inductions for a total of four full iterations of a test-learn cycle. This study is thus a proof-of-principle, implementing two possible solutions to complex optimization problems. It tests a machine learning algorithm against a baseline (random search), utilizing a robotic platform for autonomous and automated data generation and interpretation. It is meant as an orientation for researchers wanting to integrate machine learning and lab automation into their workflows to optimize complex biological problems.

## 2 Materials and methods

Details on the used lab ware and media compositions can be found in the supplementary data. Unless otherwise stated, chemicals and consumables were purchased from Analytik Jena AG (Jena, Germany), Carl Roth GmbH and Co. KG (Karlsruhe, Germany), Greiner Bio-One GmbH (Frickenhausen, Germany), Eppendorf AG (Hamburg, Germany), Merck KGaA (Darmstadt, Germany) and Sarstedt AG and Co. KG (Nümbrecht, Germany).

### 2.1 Robotic platform

Cultivation of the bacteria took place in 96-well flat-bottom microtiter plates (MTP) inside a specialized robotics platform (Model Number: 30–4448–010–26) custom build by Analytik Jena, which was hosted at the Technical University of Darmstadt, Germany.

#### 2.1.1 Hardware

**FIGURE 1:**
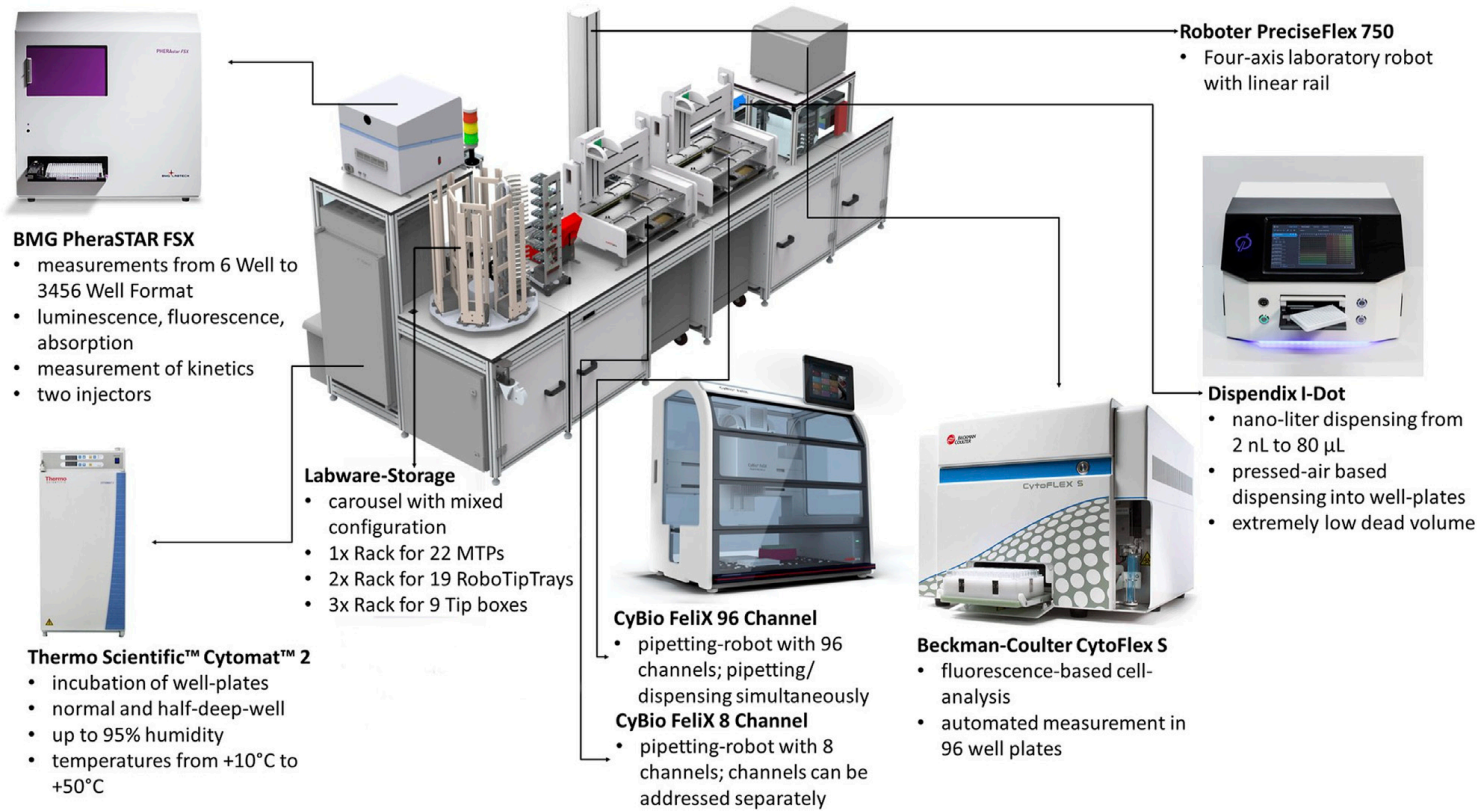
Rendering of the utilized robotic platform constructed by Analytik Jena AG including description of its major components. Cultivations were carried out in the Cytomat two incubator, pipetting operations using the CyBio FeliX liquid handlers and measurements with the PHERAstar FSX Plate reader. Not labeled are further (cooled) storage positions and the de-lidding stations.

The robotic platform incorporates different work stations for incubation, liquid handling, measurement, storage and moving plates. A rendering of the robotic platform is shown in Figure 1. The following modules of the robotic platform were used during the experiments:

• For incubations a Cytomat two tower shake incubator (Thermo Fisher Scientific) capable of incubating 29 MTPs at the same time. Plates were incubated at 37°C at 1,000 rpm.

• For measurements a PheraSTAR FSX plate reader (BMG), to measure the OD_600 nm_ and the fluorescence resulting from production of GFP in the wells of a MTP.

• For liquid handling two CyBio FeliX (Analytik Jena) liquid handling robots. *Felix1* is an 8-channel liquid handler, which pipetting volumes can be individually adjusted per well. *Felix2* is a 96-channel liquid handler able to pipette a full 96-well plate at the same time. It’s channels cannot be individually addressed.

• A linear axis mounted robotic arm with a gripper (PreciseFlex; RoboDK), transporting MTP between different work stations. The gripper also delivers new pipetting tips to both liquid handlers and retrieves used tip boxes for disposal.

• For storage multiple racks and carousels capable of storing plates, tip boxes and tip racks for both liquid handling robots. Two refrigerated positions (4°C) are also available for reagent storage in MTPs.

• A de-lidder to remove lids from plates before transferring them to measurement or liquid handling workstations.

#### 2.1.2 Software

**FIGURE 2:**
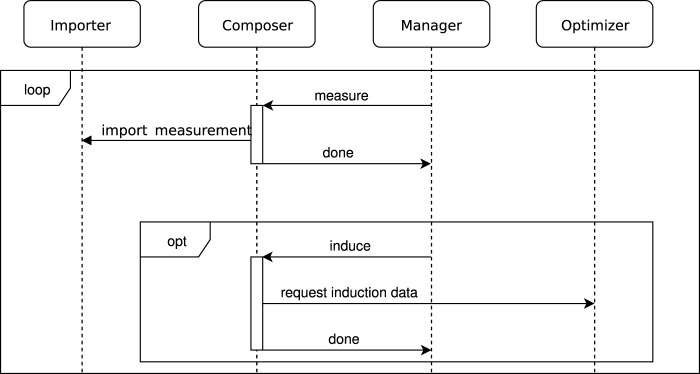
Sequence chart showing the communication between the proprietary *CyBio Composer Software*, the *Importer*, the *Manager* and the *Optimizer*. Communication between these parts is achieved via a database server which for the sake of clarity is not shown. The *CyBio Composer Software* communicates with the different machines in the robotic platform via specific, proprietary drivers. The *importer* fetches the measurement data from the plate reader and writes them to the database. The *manager* decides which of the following jobs should be done next: measuring, inducing and cloning, or waiting. The *optimizer* decides which points should be evaluated next by adjusting the pipetting volumes of the liquid handling stations.

The experimental workflow is managed through a dedicated software, including a specialized *manager* software module within the platforms own *CyBio Composer Software*. The *CyBio Composer Software* module retrieves the next set of measurement points from a database, which are previously written by the *optimizer* module. The *optimizer* module, in turn, obtains measurement data from the *importer* module. This configuration enables the system to select measurement points that are of particular interest based on the objective function. All these components communicate with each other via a common database. For a more detailed understanding of how these modules interact, please refer to the sequence chart provided in Figure 2. The default file format used in the programming of the platform is a binary file which can not be easily tracked by a version management system. For this reason another supported file format which is XML based was used. This format is also more suitable than the binary format for storing in a version control system. Version control was done using GitLab.

### 2.2 Cultivation

#### 2.2.1 Precultures

For biological system 1, *Bacillus subtilis* strain PY79 was used as a strain background to generate strain S11142, expressing GFP under control of the lactose/IPTG inducible promoter hyper-spank (see Table 1). Strain S11142, grown on LB agar plates (NaCl 5 g/L) containing 20 μ g/mL Zeocin, was used to inoculate a 200 mL shake-flask with 20 mL of MSM media supplemented with 1% glucose and 0.25% Casein acid hydrolysate. After 16 h of incubation in a shaking incubator (New Brunswick Innova 44 - Eppendorf AG, Hamburg, Germany) at 37°C and 200 rpm, the culture OD_600 nm_ was adjusted to 0.1 by diluting in appropriate amounts of fresh media and antibiotic. The adjusted culture was distributed to a MTP, filling up rows B to G with 200 μ L/well of culture. Four more MTP were filled in this layout, containing 180 μ L of sterile media in the aforementioned wells instead of bacterial culture. For all five prepared MTP, row A and H were filled with sterile media to serve as a blank, cross contamination control and to protect against evaporation during cultivation. For the latter reason wells 1 + 2and11 + 12 in rows A + H were filled with sterile, double distilled water (ddH_2_O).

**TABLE 1:** Bacterial strains and plasmids used and generated in this study.

ID	Organism	Genotype or relevant characteristics	Source
S11142	*B. subtilis* PY79	*amyE::* ( ${\text{P}}_{hyperspank}-GFPmut2,$ ${Spc}^{R}$ )	This work
		Δ *sigF::lox72,* Δ *skf::Spc* ^R^	
S29013	*E. coli* BL21 + pBS682	fhuA2 [lon] ompT gal [dcm] δ hsdS	This work
pBS682	Plasmid	PT5-lacO_GFP	

Experiments for biological system two were carried out with *Escherichia coli* strain BL21 (DE3), transformed with plasmid pBS682 yielding strain S29013. Plasmid pBS682 consists of an SC101 backbone, carrying a GFP which is controlled by a T5-lacO promotor inducible by lactose and IPTG. For cultivation during robot experiments, a modified M9 Media was used, which was supplemented with MOPS buffer and EnPump polymer (EnPresso, Berlin; see Media and Supplements) as a carbon source. The polymer enables controlled glucose release by adjusting the amount of added enzyme. Strain S29013 grown on LB agar plates (NaCl 10 g/L) containing 100 μ g/mL ampicilin was used to inoculate 20 mL of EnPump media in shake flasks. After 16 h of incubation at 37° C and 200 rpm, OD_600 nm_ was adjusted to 0.1 by diluting in appropriate amounts of fresh EnPump media containing 100 μ g/mL ampicilin. Culture and media plates were prepared as described earlier, but with EnPump media instead of MSM-Glc media. Pipetting operations to prepare 96-well plates for all experiments in the robotic platform were carried out using an epMotion 5,075 liquid handling robot (Eppendorf AG, Hamburg, Germany).

#### 2.2.2 Cultivation in robotic platform and experimental workflow

**FIGURE 3:**
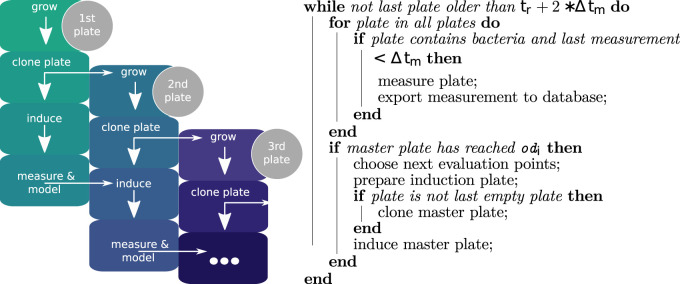
Left: Iterative work flow performed by the robotic platform: The master plate is inoculated manually as described. All further steps are autonomously performed by the robot based on measured values. Induction volumes are determined through models based on the previously measured plates. Right: Overview of the plate handling workflow. In the clonemasterplate step a small amount (20 μ l) of liquid is pipetted from the master plate into one of the media microtiter plate to inoculate a new plate. In the prepareinductionplate phase, the 8-channel pipetting robot fills the wells of a new empty plate with water, glucose releasing enzyme and inducer in a ratio determined by the applied algorithm. In the inducemasterplate phase a plate-operating liquid handling robot is used to transfer 20 μ l from the induction plate into each well of the cultivation plate. $\Delta t_{m}$ describes the incubation period between two measurements. $t_{r}$ describes the time after the induction happened, this variable is relevant for the evaluation of $gfp\left(x\right).{od}_{i}$ describes the OD at which the biological system should get induced.

At the beginning of the workflow, the robot platform was loaded with a microtiter plate containing cultures at OD_600 nm_ 0.1 as well as four additional MTP containing sterile media. Appropriate numbers of tip boxes and racks for the liquid handling workstation were added, along with the necessary reagents and empty MTP to mix together the inducer mix when needed. These reagents were sterile water and 20% (w/v) lactose in tubs placed directly at the working positions of the 8-head pipetting robot *Felix1*, as well as diluted enzyme (ENPump 2000 Reagent-A diluted 1:2 in sterile ddH_2_O). The microtiter plate containing the initial cultures is referred to as the master plate. Immediately after starting the experiment, it is transferred to the incubator. Cultivation of MTP in the robotic platform took part in a Cytoma t2 automated incubator (ThermoFischer) at 37° C and 1,000 rpm. All plates are identified with a unique barcode, which allows tracking plates throughout the whole workflow. Every hour, the cultivated plates were transferred from the incubator to a Pherastar FSX platereader (BMG Labtech), where OD_600 nm_ and fluorescence (excitation 485 nm, emission 520 nm) were measured. The measured GFP is converted to absolute fluorescence according to the iGEM standardization protocol provided at protocols.io (# 6zrhf56). The calibration data is provided in the Zenodo repository referenced in the Data Availability section. Once the average OD_600 nm_ of a plate across all cultivated wells reached 0.6 the induction process was started (Figure 3). The process consists of three different stages, each carried out by one of two CyBioFelix pipetting robots. In the first stage, an induction mixture was prepared. For this,the enzyme solution storage plate was transferred to the deck of *Felix1* from cooled storage, together with an empty 96-well V-bottom microtiter plate. In the empty plate a mixture of inducer (20% lactose or 20 mM IPTG), ddH2O and enzyme solution (in biological system 2, responsible for glucose release) was prepared to a total volume of 95 μ L. For the single parameter optimization (System 1), the maximum inducer volume in the mixture was only limited by the maximum total volume of the induction mix, resulting in a maximum IPTG concentration in the cultivation well of 2 mM. For the two-parameter optimization (System 2), a maximum of 75 μ L of 20% lactose solution and 20 μ L enzyme solution (1,500 U/L) were mixed, while the remaining volume was made up of sterile water. This resulted in a final lactose concentration between 0 and 43.8 mM in the cultivation wells and a maximum enzyme concentration between 0 and 30 U/L. Each components volume was determined by one of the two algorithms explained under Section 2.3. For biological system 2, the enzyme storage plate was transferred back to the cooling position after preparing the induction mix and the premixed induction plate was transferred to the *Felix2* deck. The cultivation plate which had reached OD_600 nm_ 0.6 was transferred to the 96-Well *Felix2* deck from the incubator as well as a sterile microtiter plate containing 180 μ L fresh media/well. The 96-channel *Felix2* was used to transfer 20 μ L of bacterial culture from each well of the cultivation plate to the fresh plate, resulting in a final volume of 200 μ L per well. In the third stage, a set of fresh tips was used to pipette 20 μ L from the premixed induction plate into the old culture plate, bringing back its volume to around 200 μ L. By using the prepared induction plate to induce all wells at the same time, in contrast to pipetting the inducer directly into the master plate, three things were achieved: Firstly the amount of time the master plate is not incubated and agitated at 37°C is reduced. This is necessary since the physiology of the cultivated bacteria can react to changing environments within a very short time and adjust its gene expression patterns accordingly. Secondly a higher precision of the induction volume is achieved, since the error of the *Felix1* liquid handling robot is larger for small volumes. Thirdly induction for all plates happens at the exact same time, reducing intra-plate variations. Both MTP, one being newly inoculated and the other containing the induced culture were then transferred into the incubator and measured for OD_600 nm_ and fluorescence every hour. Once average OD_600 nm_ of the newly induced plate reached 0.6, the induction process starts anew until all iterations were induced (Figure 3). For the last plate, the 20 μ L usually used to inoculate a new plate were discarded. The data recorded 4 h after the induction was then used by the optimization algorithm to assess the biological systems response to different inducer (and enzyme) concentrations. Depending on the algorithm running, new points of interest along the response curve were then defined, determining the amounts of inducer and enzyme added for induction of the next plate. The experiments thus was ended 4 h after the induction of the last plate once the last measurement was taken.

### 2.3 Algorithm and optimization

The aim is to find the optimal inducer concentration for a given system. In the simplest form, this would be defined as the highest fluorescence output (GFP production). Without attributing a cost factor to the inducer, the platform would answer this question simply with adding the maximum amount of inducer unless effects like arising toxicity would lower protein expression at higher concentrations. Since the amount of inducer is an actual cost driver in industrial scale production, an arbitrary inducer cost is attributed to the inducer, so the system has an optimum where a high production of the target protein (in this study GFP) can be produced with a low inducer input x .

$$f\left(x\right)=\alpha_{i}x+\alpha_{g}gfp\left(x\right)$$ (1)

In Function 1, $\alpha_{i}$ is a negative value describing the price for the inducer and $\alpha_{g}$ a positive value describing the price for the GFP output. gfp(x) is a function describing the GFP output of *B. subtilis* for an given inducer input x . This function gfp(x) can be evaluated on the robotic platform, but is noisy and slow to evaluate, since in contrast to simple mathematical functions, experiments with the described biological system take hours to evaluate. Defining x=0 for no inducer and x=100 for the maximum inducer concentration allows us to use bounded optimization algorithms.

Since we are dealing with a bacterial expression system, the system has inherent noise both from extrinsic factors such as imperfect uniformity of physical parameters across the plates as well as from intrinsic, cellular factors (Pal and Dhar, 2024) influencing gene expression, meaning that even if multiple wells are induced with the same x , the resulting fluorescence and optical density measurements will vary from experiment to experiment. Effectively this results in an inter-plate variety, which is sometimes larger than the intra-plate variety between different wells with different inducer concentrations. The effect is more pronounced between non consecutive plates, e.g., plate 1 and 4, rather than plate three and 4. Intra-plate variability is handled by GPR as conflicting evidence (stochastic process), this results in an increased uncertainty and a shift of the expected cost in the measured point. To deal with inter-plate variety, a second axis was introduced describing the distance between plate measurements. This allowed the algorithm to assign less weight to older measurements, prioritizing more recent results when evaluating future points of interest.

#### 2.3.1 Gaussian process regressor (GPR)

Gaussian Process (GP) is a method to model hidden functions from a given prior and from observed data (Rasmussen and Williams, 2005). The output of the Gaussian Process is the distribution function. The acquisition function describes which point to sample next by weighing between exploration of areas of high uncertainty and exploitation of areas with high values and low uncertainty. The Upper Confidence Bound (UCB) acquisition function (see Function 2.3.0.1) prioritizes points with high mean prediction μ and a high uncertainty σ . The parameter κ trades of exploitation vs. exploration.

$$UCB\left(x\right)=\mu \left(x\right)+\kappa \cdot \sigma \left(x\right)$$

The Expected Improvement (EI) acquisition function (see Equation 1) points that could result in a new best value.

$$EI\left(x\right)=E\left[max\left(0,f\left(x\right)-f\left(x^{⋆}\right)\right)\right]$$

The standard GPR process would be to train a GP on the previous samples and use the acquisition function to find the next sampling point. Since a parallelized approach in microplates is used, multiple points have to be sampled at the same time.

For this, the algorithm selects one sampling point, adds this point to the set of points that are induced in the next plate, and adds this point with the median as an observed point to our list of known values. It then build a new GPR, selects a new point with the acquisition point and repeats this process until enough distinct sampling points have been gathered. For the UCB we increase set κ to 20∗κ to use higher values of exploration in later and uncertain GPR. This process could fail and result in no new values, in this case values surrounding the last value are added as a fallback. For the first plate, where no points where yet sampled the points are selected randomly. The above described algorithm does not yet incorporate the previously described plate related noise. All other not systemic types of noise can be corrected for by the GPR. The GPR does not require an outlier detection preprocessing (Rasmussen and Williams, 2005). To handle inter-plate noise we introduce the plate count as a new axis to the GPR hidden function. Changing it from f (inducer) - > x to f (inducer, plate) - > x. The GPR model then can be used to predict the hidden function for a specific plate.

Alternatives to the chosen GPR exist. Neural Networks, for example, can be used to solve the bounded optimization problem. This could be especially useful if enough training data exists to correct noise introduced by well position (i.e., corner or edge wells). Yet this method would be significantly more complicated and it is still an open question to create explainable Neural Network. Furthermore are most Neural Network frameworks not specifically designed for probabilistic regression tasks. The utilization of decision trees as an alternative is suboptimal for regression tasks and would require additional modeling for this problem. Gaussian Process Regression (GPR) effectively handles uncertainties in training data, producing a model that explains the data and is inherently interpretable. GPR was also chosen because it requires only weak assumptions, for example, that the hidden function is continuous. That is a safe assumption for growth curves. Linear models can only be used with an appropriate kernel to correct for the non linearity of the hidden function, which would introduce new assumptions and complexity. Last but not least with scikit-learn a robust GPR implementation exists.

#### 2.3.2 Software for learning algorithms

For importing, analyzing and optimizing measurement data, python 3.7 was used with the following packages: *joblib* version 0.13.2 for model export, *matplotlib* version 3.1.1 for plotting, *pymssql* version 2.1.4 for communication with *Microsoft SQL Server Express* version 12.0.2269.0. *scikit-learn* version 0.21.3 for their Gaussian process regression implementation (Pedregosa et al., 2011), *scipy* version 1.3.1 for statistical functions (Virtanen et al., 2020), *plotly* multiple version for plotting.

## 3 Results and discussion

### 3.1 Biological system 1: cultivating *Bacillus subtilis* for data generation, single parameter optimization and algorithm selection

**FIGURE 4:**
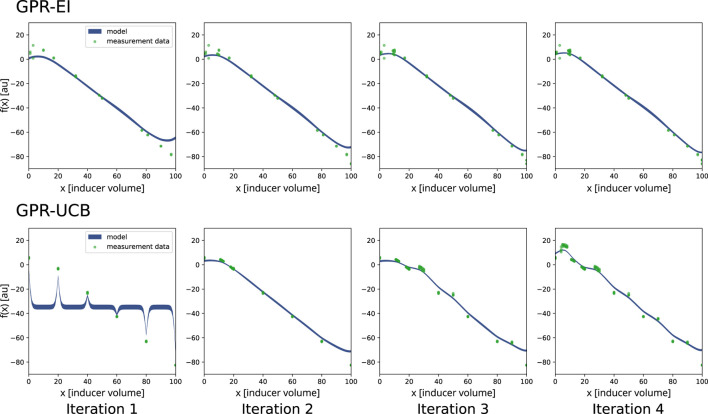
Comparison of the two search algorithms selected for a single-parameter optimization on the robotics platform. The chosen algorithms were Gaussian process regression with expected improvement (GPR-EI) and Gaussian process regression with upper confidence bound (GPR-UCB). For both experiments, the four consecutive plates which were induced as determined by the algorithm are shown in order left to right. The green dots represent a portion of the measured data for the respective plate. The blue line is the Gaussian process regression (GPR) of the search algorithm. The thickness of the blue line describes the model variance. f(x) on the *y*-axis is the objective function. 20 mM IPTG were used as inducer and mixed with ${\text{ddH}}_{2}$ O to a total volume of 100 μ L. 20 μ L of this mixture were then used to induce the corresponding cultivation well, resulting in final inducer concentrations ranging from 0 to 2 mM.

For the first system, two algorithms, namely, Gaussian process regression with expected improvement and a double Gaussian process regression with upper confidence bound were chosen to be implemented to optimize the inducer (IPTG) concentration and compared with random search (Rasmussen and Williams, 2005). The software development aim of this first set of experiments was to show a functioning integration of data analysis and experimental planning of an ongoing experiment on the robotics platform. Another aim was to evaluate two different optimization algorithms on a basic, single parameter optimization to compare the different algorithms for future multi-parameter optimizations. Two experimental runs were conducted for each algorithm (Figure 4), as well as two runs employing random search (Supplementary Figure S1).

Inducer amounts (IPTG) differed for each well and were chosen either by the optimization algorithm, or randomly generated numbers, depending on the algorithm controlling the respective experiment. Since only the inducer amounts were varied, this approach presents a one dimensional optimization problem. A range of 0 mM–2 mM of final inducer concentration in the wells was investigated. The experiments confirmed even growth across the plates and the possibility to repeatedly inoculate a new plate from the previous one.

Figure 4 shows a comparison of both algorithms. A measurable influence of IPTG induction onto measured fluorescence can be seen. Due to the cost factor attributed to IPTG (Equation 1), the optimum of the objective function f(x) is not located at the highest measured fluorescence, but rather at the highest ratio between IPTG usage and fluorescence output. This optimum for the experiments lies within a target range between 7 and 9% IPTG solution (20 mM) in the induction mix, resulting in a final IPTG concentration between 0.14 and 0.18 mM in the cultivation. In the single parameter optimization, random search evaluated the target range six times during the first run and eight times during the second run. GPR-UCB evaluated the optimal target range [7,9] 16 times on the first run and 8 times in the second run. GPR-EI evaluated the target range 16 times on the first run and 44 times in the second run. The GPR-EI was chosen because it performed marginally better, since it evaluated the target range more often. It was then compared to random search in a two parameter optimization experiment by growing *E. coli* in a glucose limited fed-batch incubation using lactose to induce GFP production as described below.

### 3.2 System 2: application of GPR-EI for 2-parameter optimization

In the second system, the previously chosen optimization algorithm GPR-EI was compared with a random search algorithm. For this, *E. coli* was cultured in M9-ENpump media. Variables for this system were the inducer (lactose) concentration as well as the amount of added enzyme. The added enzyme degrades the ENpump polysacharide, releasing glucose at an adjustable rate in the process, thus allowing to control growth rates. (Krause et al., 2016). While glucose can provide additional energy to the cells, its presence also inhibits the lactose inducer as well as lactose uptake into the cell (Inada et al., 1996). Furthermore excess glucose can results in overflow metabolism, which is an unfavorable state in *E. coli* protein production as it, for example, leads to acidification and a waste of energy in order to regenerate NAD+. (Rosano and Ceccarelli, 2014). A total of three experiments with *E. coli* were conducted, one using random search and two using the optimization algorithm GPR-EI.

**FIGURE 5:**
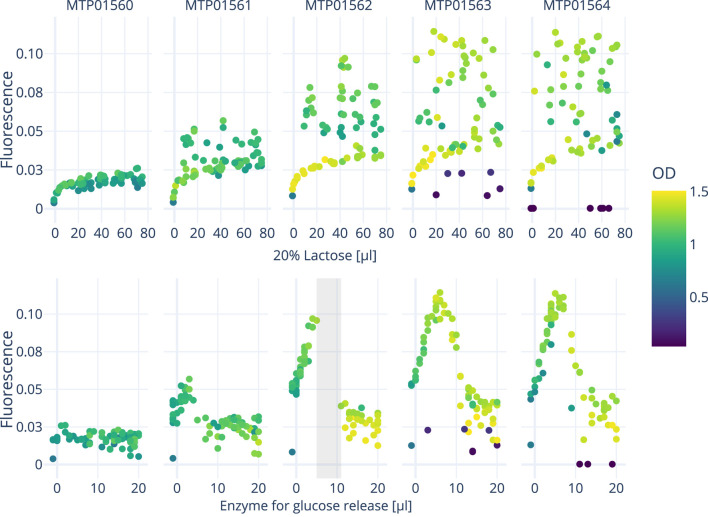
The graph displays the response curves of all five plates from a single experiment conducted on the robotics platform. These curves represent the effects of varying concentrations of lactose (inducing GFP expression) and enzyme addition onto fluorescence and OD. A surrogate-based optimization using a Gaussian process model with an expected improvement acquisition function was employed to determine the next points of interest.

**FIGURE 6:**
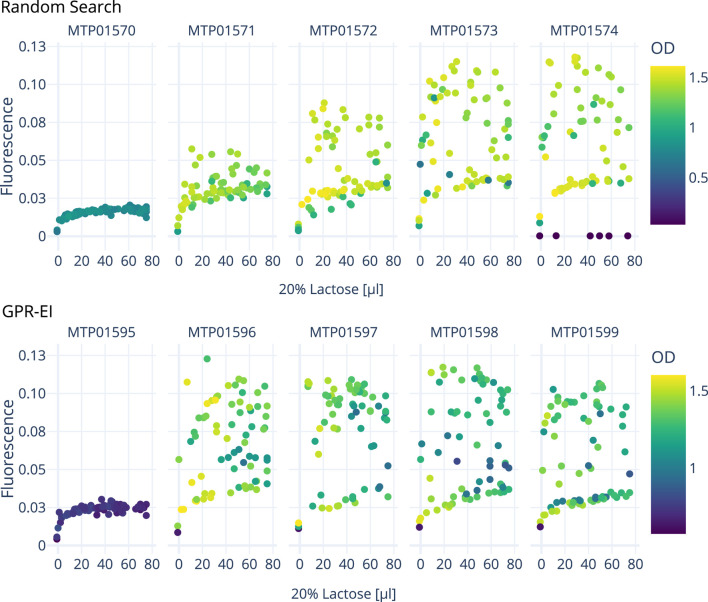
Comparison of two *E. coli* cultivation over 5 consecutive 96-well plates carried out on the robotics platform to optimize GFP production by varying the amount of inducer (lactose) and enzyme added resulting in glucose release. A random search approach is compared with a Gaussian process model with an expected improvement (GPR-EI). Shown are the measured fluorescence and OD values 4 h after the induction of each plate in response to different lactose concentrations. An interactive 3D plot is provided as a supplemental file.

Figures 5, 6 show the growth characteristics (OD_600 nm_ and measured fluorescence 4 h after induction) of different plates over the course of the three experiments. In all cases, the master plate shows lower average ODs (around 0.7–0.9) in comparison with the iterations inoculated later (Plate 1–4; between 1.0 and 1.4). For plates 1-4, OD_600 nm_ values up to OD_600 nm_ 1.6 can be observed. Cultures in the master plate were cultivated for over 20 h before reaching the induction OD_600 nm_ of 0.6. Fluorescence and OD_600 nm_ show higher degrees of variance after the induction for plates 1-4, likely due to the cultivated bacteria adopting to the cultivation conditions. These differences highlight the importance of the initial master-plate as a quasi pre-culture, ensuring adequate adaptation and growth before subsequent measurements, while diminishing in relevance in later iterations due to the inter-plate noise mitigation described in Section 2.3.

#### 3.2.1 Determination of inducer and glucose release effects on GFP expression

**FIGURE 7:**
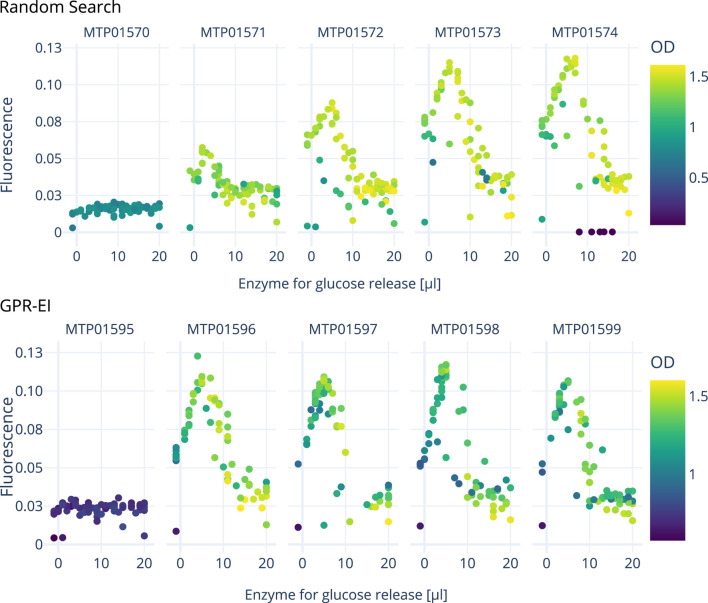
Comparison of two *E. coli* cultivations over 5 consecutive 96-well plates carried out on the robotics platform to optimize the amount of inducer (lactose, inducing GFP expression) and enzyme (resulting in glucose release). A random search approach is compared with a Gaussian process model with an expected improvement (GPR-EI). Four hours after each induction, the measured data shown here was processed to determine optimal inducer and enzyme concentrations for the following plates induction. An interactive 3D plot is provided as a supplemental file.

Measurements for OD_600 nm_ and fluorescence 4 hours after induction were used for data analysis by the active learning algorithm. The amount of added enzyme and thus available glucose in the cultivation has a distinct effect on the measured fluorescence (Figure 7) with an optimum between 7–9 μ L enzyme solution added to the induction mix. This is equal to a final concentration between 11 and 14 U/L in the cultivation well. Higher volumes of added enzyme have a negative effect onto fluorescence, likely due to glucose repression of the lac-operon controlling GFP expression. As illustrated in Figure 6, the influence of varying lactose concentrations on GFP expression becomes less pronounced at higher levels. While the absence of lactose results in minimal fluorescence, a sharp increase in fluorescence until the addition of approximately 20 μ L of lactose to the induction mix can be seen, corresponding to a concentration of 12 mM in the media. Beyond this point, baseline fluorescence starts to plateau with further additions of lactose. The notable variance in fluorescence measurements when plotted against the volume of lactose added can most likely be attributed to the concurrent effect of varying volumes of glucose releasing enzyme in the induction mix. This becomes especially apparent when looking at the interactive 3D plots provided as a supplemental file, displaying the combined effect of inducer and enzyme amount onto fluorescence and OD_600 nm_. As the experiment progresses, the algorithm seeks to sample areas with either a high potential of containing a local optimum, or which harbor a high amount of uncertainty (exploration vs exploitation). This in turn also leads to areas not being sampled if the algorithm deems them to be of little interest to these two objectives. One such areas is marked grey in Figure 5.

## 4 Lessons learned

Custom build robotic platforms like the one used in this workflow are not “off-the shelf” products which to a large degree hinders direct reproduction by others. While research communication exists to help decide whether establishing automation in ones lab is a recommended approach (Rupp et al., 2024) the potential for usage is wide, ranging from assisting researchers in a single specific task (Level 1) all the way to full automation (Level 5) where human interaction is not required under any circumstances (Stephenson et al., 2023). Because of this, we aim to show the potential of such workflows, inspire further research and disseminate our lessons learned during implementation of the workflow. Setting up a working, feed-back-loop process on a robotic system has many pitfalls and in the following subsection we share some we experienced.

### 4.1 Versioning of workflows to track changes

Many iterations of workflow instructions have to be tested, retaining the working ones while pruning those that cause failures during the robot run. Over the course of developing and advancing such workflow, errors or unused code will interfere with previously working code, if no proper version control system, such as git, is used (See Materials and Methods). This requires the vendors operating software of the robot to be in a non-binary format, optimally a popular programming language such as python or at least a structured markup languages such as XML.

### 4.2 Chose a modular approach

Due to the expensive nature of consumables, reagents and runtime, a modular approach to generating a complex process should be chosen to limit failed runs. We found it helpful to first show the function of individual operations, e.g., getting a plate from the incubator to microplate reader and measured, then a single full iteration (inoculation, measurements, induction, measurements) and only then a full, complex workflow.

### 4.3 Test the workflow in a run using colored dyes

Molecular biology frequently involves transferring small amounts of clear solutions. While an experienced human operator is able to check his operations visually, robotic platforms have no eyes. Thus running the workflow with dyes can help to visualize and measure error as well as contamination sources. When pipetting the inducer, for example, the pipette tips went too deep into the stock solution, transferring incorrect amounts of inducer due to droplet buildup on the outer surface of the pipette tips. This became apparent through appropriate controls and could be troubleshooted using fluorescein solutions as an readily quantifiable compound.

### 4.4 Randomize to tackle plate effects

While randomizing the positions of individual wells on each plate, instead of sorting them, e.g., by inducer amounts is an efficient way to tackle plate specific positioning effect (Roselle et al., 2016), we found that position specific effects also exists in other places of the workflow. Evaporation in the incubator, despite being a humidity controlled environment, as well as in the plate reader during measurements can effect plates and wells within differently. Keeping track of the exact travel paths and parking positions of each plate as well as the volumes within it can be done during test runs to discover and combat such effects further.

### 4.5 Use cameras to enable debugging of occurring problems

Experiments carried out by robotic platforms or liquid handlers still involve a certain degree of randomness, which can interrupt the experimental workflow, for example, discarded tips blocking the trash chute, thus blocking the robotic arm, equipment malfunctioning or plates being dropped seemingly random. While this usually brings the workflow to a halt at some point due to built in safety features of the platform, finding out what exactly caused this error is not possible without video recordings. We thus used the open source software ZoneMinder (https://zoneminder.com/) to record videos and allow remote monitoring using firmware modified wifi cameras (https://github.com/alienatedsec/yi-hack-v5) as well as a Raspberry pi with night vision camera (Waveshare IR-Cut Camera) placed inside the shaking incubator.

### 4.6 Use mobile alerts to inform operators

If errors occur which cannot be handled by the automation platform, the running workflow will be stopped. This often results in experiments failing, as the biological systems do not stop. It is therefore beneficial to be notified of such stoppages as timely as possible. This can, for example, be achieved by sending out email or SMS alerts to qualified personnel, in case that the robot management software has not responded for more than 10 min.

### 4.7 Try to minimize user input and interaction

One consistent source of errors occurring in the workflow was wrong initial user input. This could be in the form of physical labware being misplaced or miscounted before starting the workflow, leading to the robotarm trying to retrieve labware from an empty slot, dropping labware onto an already occupied slot or grabbing a stack instead of a single plate or pipette rack. We found that having a four eye principle when starting up the robot, with the second person potentially watching remotely with the help of installed cameras reduced the risk of such errors. Another solution, already employed by a number of small scale liquid handling platforms is the automated detection of labware using cameras. A third solution employed at this platform was the use of small lasers on turrets able to pinpoint the location selected labware had to be placed (Suleiman et al., 2024) which greatly reduced the loading time and error rate of misplaced labware.

## 5 Conclusion

In this study we demonstrate a robotics platform which can autonomously optimize multiple parameters over a continuous series of cultivations. We developed a software framework enabling the presented system to inoculate new plates from previous cultivations, measure growth and expression characteristics and analyze the gathered data without user input. Using a search algorithm, the system is able to determine points of interest for induction of subsequent cultivations. The system mixes the inducers for each well independently, retrieving the necessary data from a database. Usage of this database enables the workflow to be adapted to compute measured data in different ways and serves as the central hub for data. The digital documentation of all pipetting volumes and measured datapoints provide a high reliability and traceability compared to a human operator. This provenance, combined with the autonomous optimization greatly enhances the walk-away time of human operators since the robot can work unsupervised after being loaded with supplies once. The chosen expression systems exemplify two important industrial production systems. Especially the two parameter optimization represents a common combination of host (*E. coli*), promoter system (lac) and feeding strategy (glucose limited fed-batch). Consequently, the observed higher GFP expression for lower concentrations of available glucose is supported by previous observations (Neubauer et al., 1992).

While the use of a optimization algorithm allows finding optimal solutions more efficiently in respect to utilization of resources, a random search across the sample space also allows finding optima for a two parameter optimization. This shows that even lab automation without optimization algorithm based feedback loops can proof useful to reduce human intervention. Such simpler implementations can furthermore serve as a stepping stone towards higher degrees of complexity. While the chosen two parameters (inducer and enzyme added) and their effect on OD and GFP expression (fluorescence) can still be visualized and comprehended by a human operator, the presented system could be easily scaled to multi-dimensional optimizations with higher number of samples. The robotic platforms incubator used in this work, for example, holds a maximum of 29 plates, of which only five were in parallel use for this study. It has to be said though, that merely increasing the numbers of iterations and plates in parallel use is not a endlessly scalable solution. With too many plates having to be moved, inoculated and induced, scheduling issues are expected to arise, since moving plates between the different positions takes considerate amounts of time. This problem is being actively addressed in the community (Itoh et al., 2021). One alternative could be to use 384-well plates, which would address the bottle neck of transporting times per sample, yet would require more experience with the influence of well geometries on important cultivation parameters such as oxygen transfer rates. The applied methodology of Gaussian Process Regression (GPR) would struggle to scale beyond ten parameters due to its computational complexity, which is well beyond the likely limit reachable with the automation setup. Additionally, as the number of parameters increases, the kernel optimization process becomes more challenging, often leading to over-fitting or poor generalization (Rasmussen and Williams, 2005).

## Data Availability

The datasets presented in this study can be found in online repositories. The names of the repository/repositories and accession number(s) can be found below: https://doi.org/10.5281/zenodo.13837456.
