# Supplementary material for: Closing the loop: establishing an autonomous test-learn cycle to optimize induction of bacterial systems using a robotic platform
Source: Front Bioeng Biotechnol. 2025 Jan 22;12:1528224. doi: 10.3389/fbioe.2024.1528224 (PMC11795046; doi:10.3389/fbioe.2024.1528224)
Supplement: Supplementary file 1 [file DataSheet1.pdf]

Automation-aided construction and characterization of *Bacillus subtilis* PrsA strains strains for the secretion of amylases: supplemental document

November 14, 2024

Contents

1 Introduction **S1**

2 Materials and Methods **S1**

2.1 Materials S1

2.2 Media S1

3 Data Figures and Tables **S2**

3.1 Supplemental Figures S2

3.1.1 System 1 . . . . S2

3.1.2 System 2 . . . . S2

1 Introduction

This supplemental document contains figures referenced in the main manuscript as well as additional material and methods.

2 Materials and Methods

2.1 Materials

2.2 Media

Table 1: Utilized consumables for robotic platform

Name	Specification	Manufacturer
96 MTP - F-bottom	655101	Greiner Bio-One GmbH
96 MTP - V-bottom	651101	Greiner Bio-One GmbH
Lid	656101	Greiner Bio-One GmbH
TipBoxes 96	OL3811-25-637S	Analytik Jena AG
RoboTipTray 96-250µl	OL3810-25-664	Analytik Jena AG

Table 2: **Media for *Bacillus subtilis***

**Name**	**Compound**	**Amount**
MSM	K_2HPO_4	83.8 mM
	NaH_2PO_4	26.1 mM
	$(NH_4)_2SO_4$	21.6 mM
	Na_2SO_4	14.1 mM
	NH_4Cl	9.3 mM
	$(NH_4)_2$-H-citrate	4.4 mM
	$MgSO_4$	2.0 mM
	500x Trace element solution	2 mL L^{-1}
MSM-Glc	MSM with:	
	D-Glucose	1 %
	Casein acid hydrolysate	0.25 %
500x Trace element solution (for MSM)	Ethylenediaminetetraacetic acid	54.0 mM
	$FeCl_3$	61.8 mM
	$CaCl_2$	3.4 mM
	CoCl	0.76 mM
	$CuSO_4$	0.64 mM
	$ZnSO_4$	0.63 mM
	$MnSO_4$	0.59 mM

Table 3: **Media for *Escherichia coli***

**Name**	**Compound**	**Amount**
M9	Na_2HPO_4	33.7 mM
	KH_2PO_4	22.0 mM
	NH_4Cl	9.35 mM
	NaCl	8.55 mM
	$MgSO_4$	1.0 mM
	$CaCl_2$	0.3 mM
	Glucose	0.4 %
	Casein acid hydrolysate	0.25 %
	Biotin	1 µg L^{-1}
	Thiamin	1 µg L^{-1}
	100x Trace element solution	10 mL L^{-1}
100x Trace element solution (for M9)	Ethylenediaminetetraacetic acid	13.4 mM
	$FeCl_3$	3.1 mM
	$ZnCl_2$	0.62 mM
	H_3BO3	162 µM
	$CuCl_2$	76 µM
	$CoCl_2$	42 µM
	$MnCl_2$	8.1 µM
M9-EnPump	Na_2HPO_4	33.7 mM
	KH_2PO_4	22.0 mM
	NH_4Cl	9.35 mM
	NaCl	8.55 mM
	$MgSO_4$	1.0 mM
	$CaCl_2$	0.3 mM
	Casein acid hydrolysate	0.2 %
	Biotin	1 µg L^{-1}
	Thiamin	1 µg L^{-1}
	100x Trace element solution	10 mL L^{-1}
	EnPump200 powder	15 g L^{-1}
	MOPS Pufferan	41.889 g L^{-1}

Table 4: **Inducers and Antibiotics**

**Name**	**Compound**	**Final concentration**
**Inducer**		
IPTG	Isopropyl β-d-1-thiogalactopyranoside	0 - 2 mM
Lac	Lactose	0 - 43.8 mM
Reagent A (Enzyme)	EnPumP system	0 - 30 U/L
**Antibiotics**		
Amp	Ampicillin sodium salt	100 µg mL^{-1}
Zeo	Zeocin	20 µg mL^{-1}

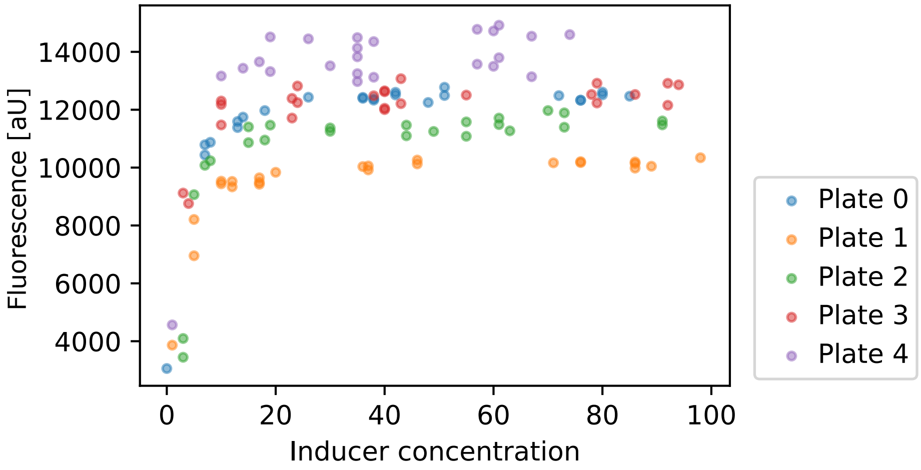

Fig. S 1: Random search algorithm applied for a *Bacillus subtilis* cultivation. The inducer concentrations (lactose) which were evaluated where chosen by random search. The consecutive plates are shown, displaying the inter plate variance.

3 Data Figures and Tables

3.1 Supplemental Figures

3.1.1 System 1

3.1.2 System 2

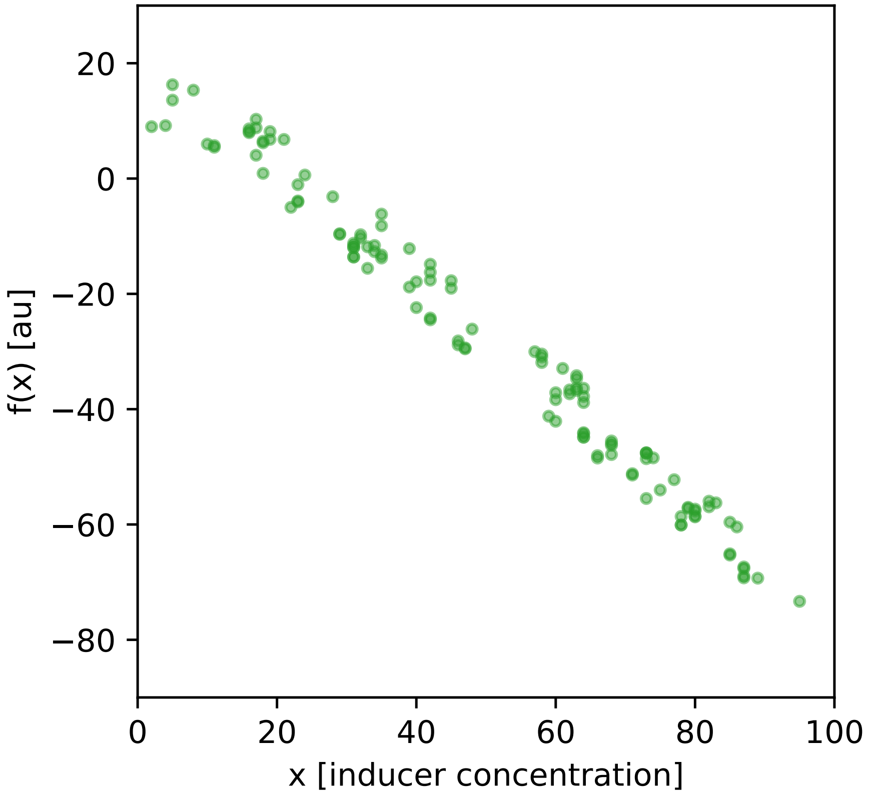

Fig. S 2: Random search algorithm applied for a *Bacillus subtilis* cultivation. The effect of different inducer concentrations is plotted against the target function, which takes into a account the produced amount of enzyme (fluorescence) as well as the cost associated with the used inducer volumes.

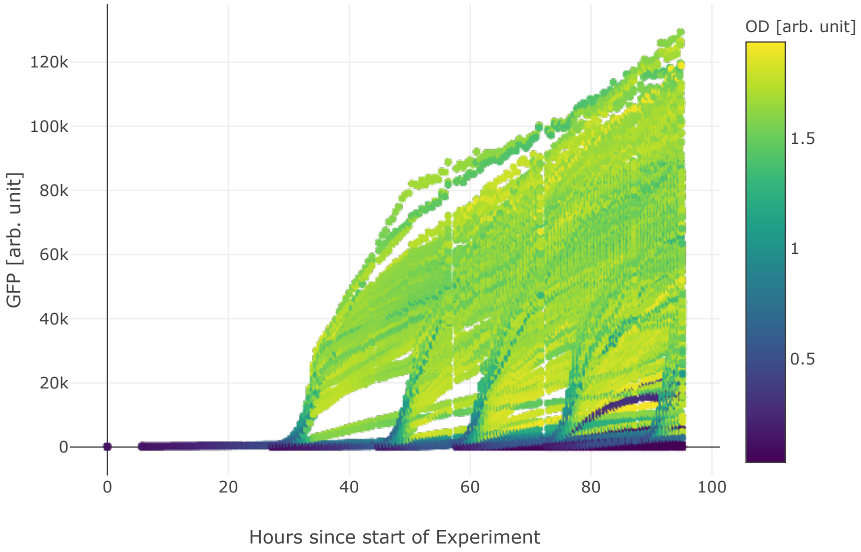

Fig. S 3: Visualisation of all measurements taken in a single experiment. As time continues (X-axis) new plates get inoculated as the previous iteration reaches and average OD of over 0.6. The effect of induction by lactose are marked by strong increases in measured fluorescence due to the expressed GFP protein.

after induction and cut after 6h

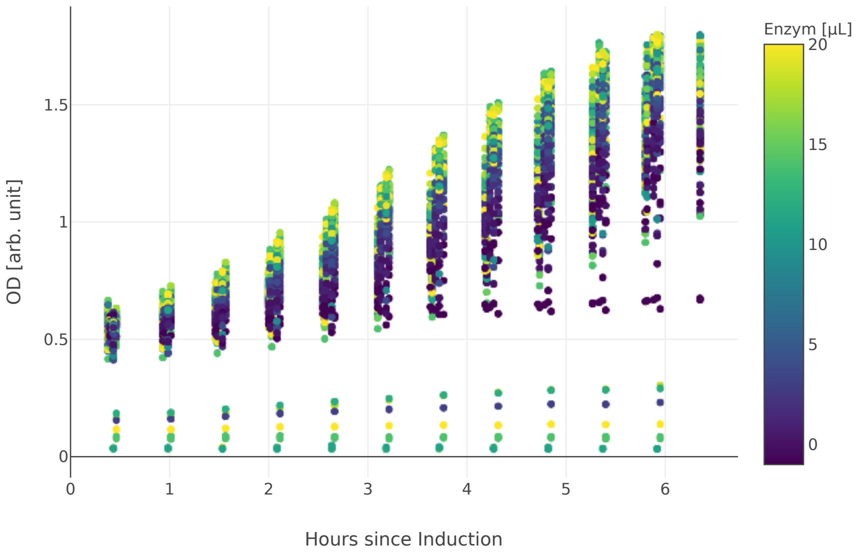

Fig. S 4: Effect of Enzyme on growth. Plotted on the X-axis is the time since induction of the measured plates. Y-axis shows the measured OD_{600nm}. The effect of lower enzyme concentrations on the growth can be seen.
